# Gestational Intraplacental Choriocarcinoma in a Term Pregnancy: A Case Report

**DOI:** 10.7759/cureus.31243

**Published:** 2022-11-08

**Authors:** Andrea Sala, Sara Ornaghi, Martina Delle Marchette, Cristina Maria Bonazzi, Sonia Gorla, Francesca Moltrasio, Robert Fruscio, Patrizia Vergani, Fabio Landoni

**Affiliations:** 1 Obstetrics and Gynecology, San Gerardo Hospital, University of Milano-Bicocca, Monza, ITA; 2 Obstetrics and Gynecology, Monza Brianza per il Bambino e la sua Mamma (MBBM) Foundation, San Gerardo Hospital, University of Milano-Bicocca, Monza, ITA; 3 Pathology, San Gerardo Hospital, University of Milano-Bicocca, Monza, ITA

**Keywords:** oncology, pregnancy, placenta, trophoblasts, choriocarcinoma, gestational trophoblastic disease

## Abstract

Intraplacental choriocarcinoma (IC) is a rare type of gestational choriocarcinoma (GC) occurring within the placenta, and only a small number of cases have been reported so far. Intraplacental choriocarcinoma is usually asymptomatic or may present with aspecific symptoms, including unexplained vaginal bleeding during pregnancy. Early diagnosis and treatment are pivotal for ensuring optimal outcomes. However, intraplacental choriocarcinoma is rarely suspected due to limited knowledge and awareness of the condition. Here, we report the case of a 34-year-old woman diagnosed with intraplacental choriocarcinoma by placental histological examination performed after delivery due to unexplained vaginal bleeding at 29 gestational weeks, requiring hospital admission. Two lines of chemotherapy and surgery were necessary to achieve complete remission. Since unexplained vaginal bleeding during pregnancy can be a clinical manifestation of intraplacental choriocarcinoma, we propose to consider placental histological examination in all pregnancies with this complication.

## Introduction

Gestational trophoblastic neoplasia is a group of rare tumors that arise from the cells of conception. The malignant forms include gestational choriocarcinoma (GC), placental site trophoblastic tumor, and epithelioid trophoblastic tumor; these forms can arise from a molar pregnancy or a normal genetic pregnancy [1].

GC has an estimated overall incidence of 1 case per 50,000 pregnancies [1]. The most significant risk factors are advanced maternal age, a previous trophoblastic disease, and Asian and South American ethnicity; nutritional deficiencies and environmental factors appear to be other less relevant factors. GC originates from the cytotrophoblast and syncytiotrophoblast cells that develop into the placenta. However, it usually becomes clinically apparent months or years after the causative pregnancy, with symptoms during pregnancy, such as unexplained vaginal bleeding, being rare [1]. For this reason, GC is frequently managed based on a clinical diagnosis without the placental histology data.

Intraplacental choriocarcinoma (IC) is a rare form of GC located within the placenta. Due to its rarity, clinical information about IC is substantially limited. Here, we present a case of IC incidentally diagnosed in a woman by a placental histological examination requested for obstetric reasons after term delivery.

## Case presentation

The patient is a 34-year-old South American woman who delivered at our hospital in October 2017 with pregestational obesity (BMI of 33 kg/m^2^). The patient's past medical history was significant for laparoscopic cholecystectomy in 2010. Her obstetric history included two abortions and two previous cesarean sections, one in 2008 for suspected fetal macrosomia and the other in 2015 as an iterative procedure.

The index pregnancy was complicated by gestational diabetes requiring dietary therapy and by an episode of vaginal bleeding at 29 weeks of gestation requiring hospital admission. Precisely, the ultrasound assessment identified a normally located placenta and a supracervical clot of 4 cm, with no clear evidence of amniochorial detachment. During the hospital stay, a transaminase elevation was identified at a routine laboratory assessment. This prompted a detailed investigation leading to a diagnosis of dysmetabolic chronic hepatopathy. The patient was discharged at 314/7 weeks’ gestation; subsequent obstetric visits were regular. She was then hospitalized again for delivery via elective iterative cesarean section at 374/7 weeks’ gestation, giving birth to a healthy female infant weighing 3310 grams. Considering the episode of unexplained vaginal bleeding requiring hospitalization and the diagnosis of maternal chronic hepatopathy, the placenta was sent for histological examination.

**Figure 1:**
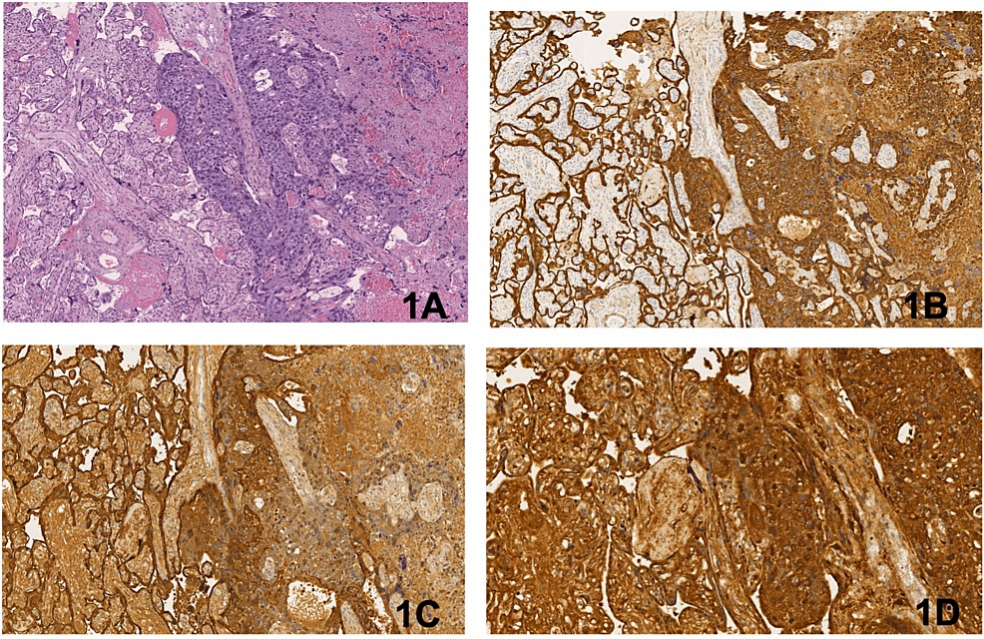
Representative images of placental histologic examination Hematoxylin and eosin stain (400×) (A), and of immunohistochemistry staining for CK (400×) (B), HPL (400×) (C), and betaHCG (600×) (D).

The macroscopic examination of the placenta identified normal amniochorial membranes and umbilical cord. The chorionic plate showed excessive fibrin deposition. Microscopically, the transition between benign and malignant trophoblasts was evident at a low power field (Figure 1A). The malignant trophoblastic cells displayed marked pleomorphism, prominent nucleoli, and frequent mitoses. Also, ischemia and infarction in the areas adjacent to the malignant trophoblastic cells were identified, likely subsequent to a compromised blood supply due to the presence of cancer cells in the intervillary spaces. Immunohistochemical staining was positive for cytokeratin AE1/AE3 (Figure 1B), HPL (Figure 1C), and beta-hCG (Figure 1D).

Upon placental histological diagnosis, the patient was recalled to undergo staging examinations. Serum hCG levels were 1109 mIU/ml on day 21 post-partum (normal levels in non-pregnant women <2 mIU/ml). The brain MRI, as well as the chest CT, did not show metastatic disease. In turn, an abdominal CT with medium contrast showed a uterus with a hyperdense alteration in the myometrium at the body-neck passage in the right paramedian site. The same uterine finding was also identified at the transvaginal ultrasound scan. The hCG levels of the newborn were negative on both blood and urine specimens.

The maternal serum hCG level was raised to 5567 mIU/ml two weeks after the initial evaluation. Thus, the patient was immediately started on chemotherapy with methotrexate (MTX) and folic acid according to ESMO guidelines [1]. Serum hCG levels declined to 8 mIU/ml after two cycles, and a hysterectomy was scheduled six days later. At the time of pre-hospitalization, the serum hCG level was found to be 55 mIU/ml. Because of this, the second line of chemotherapy was decided to be EMA-CO (etoposide, MTX, actinomycin D, cyclophosphamide, and vincristine) along with intrarachid methotrexate. This chemotherapy regimen was started five days later, with a serum hCG value of 545 mIU/ml. After two cycles of chemotherapy, the patient's hCG level dropped to 15 mUI/ml, and she underwent a laparotomic hysterectomy.

**Figure 2:**
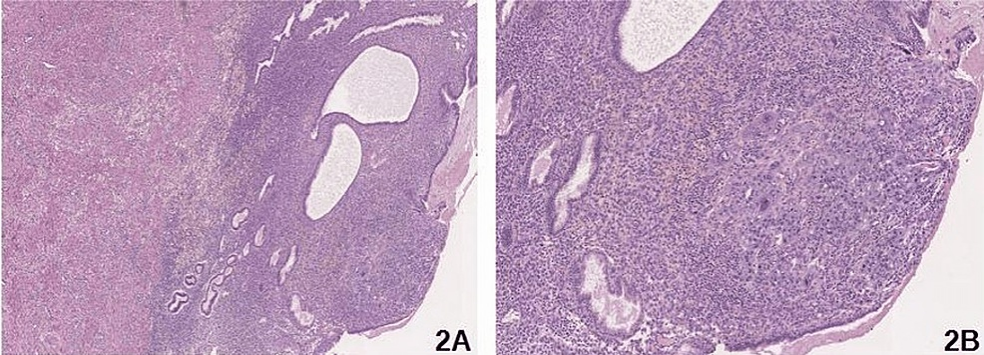
Representative images of endometrial sections from hysterectomy Hematoxylin and eosin stain: residual solid neoplastic area of choriocarcinoma in the endometrial stroma: (A) 100× and (B) 300×.

The uterine histological examination showed a minimal residual focus of pathological tissue without evidence of the infiltrative attitude of 12.6 mm × 7.9 mm at the level of the endometrium in the anterior uterine wall (Figure 2A-2B).

According to the histological examination, which indicated the persistence of active neoplastic cells at the uterine level, the patient underwent a third consolidation cycle of EMA-CO chemotherapy. The postoperative clinical follow-up was regular. Weekly postoperative serum hCG level monitoring demonstrated a complete response to treatment. The patient is currently disease-free (follow-up of 46 months); she monitors her serum hCG level every four months, and she is clinically assessed once a year at our outpatient clinic. The patient gave written informed consent for publishing this study and its accompanying images.

## Discussion

IC is a rare form of GC located within the placenta that arises from the chorionic villous trophoblast. It represents 2% of all gestational trophoblastic tumors. The clinical presentation of this tumor is very variable. It can be clinically silent or cause a metastatic maternal and/or infantile disease. The most common symptom, when present, is vaginal bleeding, either during pregnancy or afterward if uterine or vaginal metastases are present. In 50% of the cases, the diagnosis is incidental, and a subsequent pathological examination of the placenta is requested for obstetric reasons [2]. Since the placental examination is not routinely performed, detection of IC can be substantially delayed until symptoms become clinically apparent.

Feto-maternal hemorrhage, stillbirth, and intrauterine growth restriction have been reported as the most common indications for placental pathological examination among asymptomatic patients [3]. In our case, the patient was hospitalized for unexplained vaginal bleeding during the third trimester of pregnancy. During her hospital stay, she was diagnosed with chronic hepatopathy. Both conditions prompted the request for a histological assessment of the placenta, according to our institutional protocol. Of note, the macroscopic evaluation of the placenta in the delivery room, routinely performed at our center, did not reveal any suspicious lesions. In line with our observation, a recently published systematic review has reported a 29% rate of macroscopically normal placentas among IC cases diagnosed during histological placental examination [2]. Also, the authors reported that more than 50% of the IC cases showed only a single lesion, and only 10% presented multiple lesions. Usually, the primary tumor is very small, ranging from 2.5 to 8 mm, requiring an extensive pathological examination of the placenta to be found [4]. These data, along with the rarity of this condition, explain why a prenatal diagnosis of IC by ultrasound scan is extremely challenging. High levels of maternal alpha-fetoprotein in a normal fetus may raise suspicion [5].

About half of IC cases are metastatic at diagnosis because the disease is usually not considered until the mother or the child has symptoms. The most frequent metastatic sites are the lungs, the brain, the uterus, and the vagina. Notwithstanding this, the prognosis for IC is optimal, with a cure rate approaching 100%. All cases are associated with elevated serum and urine human chorionic gonadotropin (hCG) levels, which are useful for both diagnostic and surveillance purposes [2].

Our patient presented with a metastatic uterine lesion in the myometrial context, which was diagnosed early by transvaginal ultrasound and CT scan; thanks to the placental histological examination requested for obstetric reasons. Only two cases of infantile metastases have been reported, with one death and one survival after chemotherapy [2,6,7]. In our case, both blood and urinary hCG tests were negative at maternal diagnosis and at follow-up.

Given the rarity of IC, there are no specific guidelines for treatment, and those for GC choriocarcinoma are usually followed [1]. Serum hCG level surveillance is generally sufficient in cases without metastasis, and most tumors spontaneously regress. In cases of metastases, residual disease, plateauing, or rising hCG, treatment based on the FIGO score is indicated: single-agent chemotherapy (MTX) in low-risk tumors (FIGO score 0-6) and multi-agent chemotherapy (EMA-CO) in high-risk tumors (FIGO score > 6) [1]. In our case, the patient initially had a FIGO score of 3 and was treated with MTX. However, complete remission was not achieved, thus prompting a second-line multidrug regimen with EMA-CO. A hysterectomy was also required to achieve complete remission.

## Conclusions

IC is an extremely rare condition that is likely underdiagnosed. Its natural history varies, and prenatal diagnosis is particularly challenging. Early detection is possible only if a placental pathological examination is performed after delivery. However, placental histology is not routinely assessed in most hospitals. This case illustrates the importance of a detailed examination of the placenta in cases of unexplained vaginal bleeding in pregnancy and its significance in diagnosing IC. In the absence of placental histology, a post-partum serum hCG assay may be useful to raise suspicion and prompt specific investigations. The cost-benefit ratio of routine implementation of such tests should be assessed in future research.

## References

[1] Seckl MJ, Sebire NJ, Fisher RA, Golfier F, Massuger L, Sessa C (2013). Gestational trophoblastic disease: ESMO Clinical Practice Guidelines for diagnosis, treatment and follow-up. Ann Oncol.

[2] Jiao L, Ghorani E, Sebire NJ, Seckl MJ (2016). Intraplacental choriocarcinoma: systematic review and management guidance. Gynecol Oncol.

[3] She Q, Cheng Z, El-Chaar D, Luo F, Guo X, Wen SW (2018). Intraplacental choriocarcinoma coexisting with fetomaternal hemorrhage: case report, chemotherapy management, and literature review. Medicine (Baltimore).

[4] Jauniaux E (1998). Ultrasound diagnosis and follow-up of gestational trophoblastic disease. Ultrasound Obstet Gynecol.

[5] Ollendorff DA, Goldberg JM, Abu-Jawdeh GM, Lurain JR (1990). Markedly elevated maternal serum alpha-fetoprotein associated with a normal fetus and choriocarcinoma of the placenta. Obstet Gynecol.

[6] Avril MF, Mathieu A, Kalifa C, Caillou C (1986). Infantile choriocarcinoma with cutaneous tumors: an additional case and review of the literature. J Am Acad Dermatol.

[7] Liu J, Guo L (2006). Intraplacental choriocarcinoma in a term placenta with both maternal and infantile metastases: a case report and review of the literature. Gynecol Oncol.

